# Ongoing High Incidence and Case-Fatality Rates for Invasive Listeriosis, Germany, 2010–2019

**DOI:** 10.3201/eid2709.210068

**Published:** 2021-09

**Authors:** Hendrik Wilking, Raskit Lachmann, Alexandra Holzer, Sven Halbedel, Antje Flieger, Klaus Stark

**Affiliations:** Department for Infectious Disease Epidemiology of the Robert Koch Institute, Berlin, Germany (H. Wilking, R. Lachmann, A. Holzer, K. Stark);; Department for Infectious Diseases of the Robert Koch Institute, Wernigerode, Germany (S. Halbedel, A. Flieger)

**Keywords:** bacteria, case-fatality rate, enteric infections, food-borne infections, food contamination, gastrointestinal infections, Germany, incidence, listeriosis, *Listeria monocytogenes*, whole-genome sequencing, food safety

## Abstract

We used 10 years of surveillance data to describe listeriosis frequency in Germany. Altogether, 5,576 cases were reported, 91% not pregnancy associated; case counts increased over time. Case-fatality rate was 13% in non–pregnancy-associated cases, most in adults ≥65 years of age. Detecting, investigating, and ending outbreaks might have the greatest effect on incidence

*Listeria monocytogenes* infections are primarily foodborne and cause gastrointestinal disease or invasive syndromes among infected persons (*1*). Because *L. monocytogenes* is an intracellular pathogen and because invasive listeriosis is the primary manifestation in diagnosed listeriosis, persons with deficient cell-mediated immunity are at increased risk for its symptoms, including sepsis and meningitis. In addition, infection during pregnancy can lead to chorioamnionitis and fetal infection that can result in miscarriage and stillbirth even 2 months after the mother is exposed. One study found that 44% of patients with non–pregnancy-associated (NPA) listeriosis in Germany had received immunosuppressive therapy ≤3 months before illness onset and another 28% had a coexistent immunocompromising illness, such as diabetes (*2*). Testing for bacteria in blood cultures or cerebrospinal fluid (CSF) is recommended for diagnosis.

*Listeria* is ubiquitous in the environment and can produce biofilms in the food production environment and thus contaminate ready-to-eat (RTE) products, which are typically consumed raw or without further processing. *Listeria* species grow during shelf life, even at low temperatures, and multiply to concentration levels that make invasive listeriosis and outbreaks more likely. For these reasons, it is suspected that *L. monocytogenes* exposure is very common but the disease rare. However, in recent years several large outbreaks have been reported in Germany (*3*–*7*).

## The Study 

**Figure 1:**
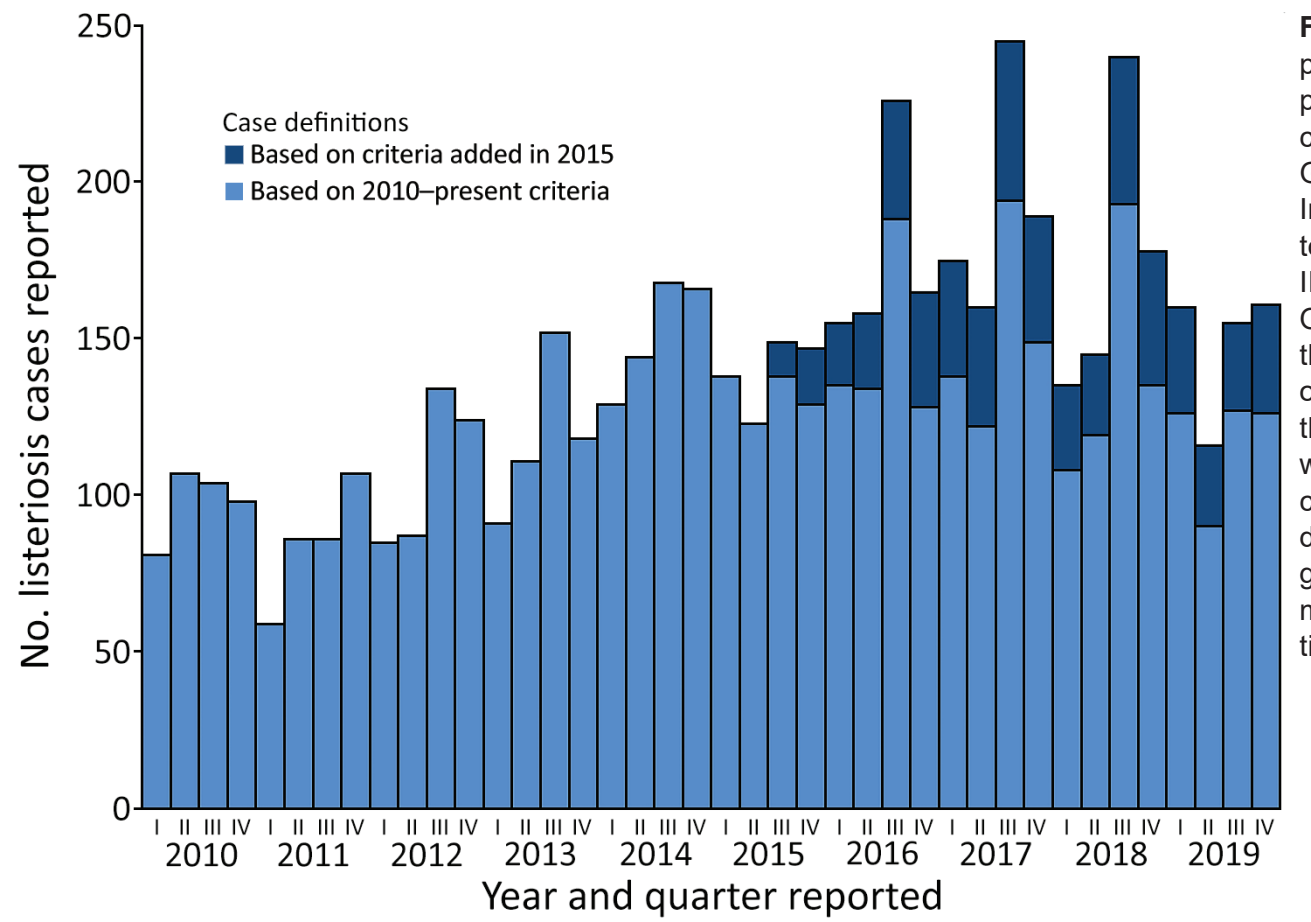
Distribution of pregnancy-associated and non–pregnancy-associated listeriosis cases, by year and quarter, Germany, 2010–2019 (n = 5,576). In the x-axis labels, I corresponds to January–March, II to April–June, III to July–September, and IV to October–December. Before the third quarter of 2015, two groups of patients were not included in the reference definition: those with unknown or unfulfilled clinical criteria and those with nucleic acid detection only. Data from these groups are displayed separately to make the changes in trends over time more apparent.

We analyzed mandatory notification data about invasive listeriosis cases in Germany during 2010–2019 to describe time trends, case-fatality rates, demographic distribution, clinical and diagnostic characteristics, and geographic trends (Appendix). In total, 5,576 listeriosis cases were reported during the 10-year study period; 5,064 (91%) of those were NPA and 486 (9%) were pregnancy associated, 241 in mothers and 245 in newborns. Information on disease manifestation was not transmitted for 26 cases. The lowest annual incidence was in 2011 (0.41/100,000 residents) and the highest in 2017 (0.93/100,000 residents); the average for 2010–2019 was 0.69/100,000 residents. We observed a steady increase in cases during 2011–2017, but incidence in 2019 was lower than in previous years. Exceptionally high numbers were reported in the third quarters of 2016, 2017, and 2018 (Figure 1).

Among the 5,064 NPA listeriosis case-patients, 2,032 (40%) were female and 3,855 (76%) were >65 years of age (Table 1). Listeriosis among adolescents and children other than newborns is rare (37 cases). Incidence in adults 18–44 years of age is <0.1/100,000 residents, in contrast with incidence among adults ≥85 years of age: 3.99/100,000 residents for men and 2.08/100,000 residents for women. Annual median age of case-patients increased steadily from 72 years of age in 2010 to 77 years of age in 2019.

**Table 1:** Average annual incidence of notified cases of non–pregnancy-associated listeriosis, by age and gender, Germany, 2010–2019*

Patient age, y	No. male case-patients	Incidence among male case-patients	No. female case-patients	Incidence among female case-patients	Overall no. cases	Overall incidence
Total	3,029	0.74	2,032	0.48	5,061	0.61
≤17	15	0.02	22	0.03	37	0.03
18–44	84	0.06	87	0.07	171	0.06
45–49	56	0.21	37	0.14	93	0.18
50–54	120	0.35	68	0.20	188	0.28
55–59	195	0.58	100	0.30	295	0.44
60–64	280	1.01	145	0.51	425	0.75
65–69	389	1.68	207	0.81	596	1.23
70–74	509	2.96	295	1.51	804	2.19
75–79	612	3.53	371	1.73	983	2.54
80–84	452	3.30	369	1.92	821	2.49
≥85	317	3.99	331	2.08	648	2.71

*Incidence is given as no. cases/100,000 residents.

**Figure 2:**
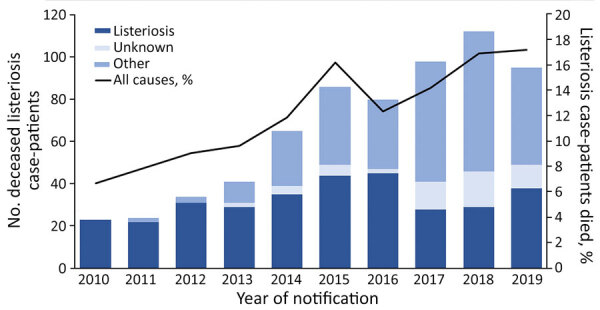
Distribution of non–pregnancy-associated listeriosis cases (n = 5,061) in which the patients died (n = 658) and case-fatalities by year and cause of death, Germany, 2010–2019. Black line indicates percentage of infected persons who died.

Sources for testing samples included CSF (657, 13%), blood (4,097, 81%), and material from other usually sterile sites (274, 5%) (Table 2). A significantly higher proportion of *L. monocytogenes* was detected in CSF among adults 18–64 years of age (24%) than among those >65 years of age (9%) (p<0.01); for most case-patients >65 years of age, the isolate was detected from blood. Most NPA case-patients (95%) were hospitalized; we found no differences among age groups (p = 0.689). Altogether, 658 NPA case-patients have been reported deceased. The case-fatality rate for NPA cases was 13%, significantly higher among patients >65 years of age (14%) than among those 18–64 years of age (10%; p<0.001). Listeriosis was the main cause of death for 324 (49%) of NPA case-patients and a contributing factor for 280 (43%). NPA case-fatality rates increased over the 10-year study period, but mainly because of an increase in listeriosis case-patients who died from causes other than listeriosis (Figure 2). For 54 (8%) deceased case-patients, cause-of-death information was missing. Of 301 pregnancy-associated cases, 50% were confirmed from blood cultures and 54% from samples of newborn, stillborn, or maternal tissues (in some cases, both). A total of 32 fetal losses and 26 neonatal deaths resulted in a case-fatality rate of 19% for pregnancy-associated cases. 

**Table 2:** Clinical characteristics of notified cases of invasive listeriosis, Germany, 2010–2019*

Characteristic	Pregnancy-associated, no. (%) cases	Non–pregnancy-associated, no. (%) cases			
		Children/adolescents <18 y	Adults 18–64 y	Adults ≥65 y	Total
Total	301 (100)	37 (100)	1,172 (100)	3,855 (100)	5,064 (100)
Sex					
F	301 (100)	22 (59)	437 (37)	1,573 (41)	2,032 (40)
M	0				
Isolate source†					
Cerebrospinal fluid	6 (2)	21 (57)	277 (24)	359 (9)	657 (13)
Blood	152 (50)	15 (41)	800 (68)	3,282 (85)	4,097 (81)
Other sterile site	NA	1 (3)	87 (7)	186 (5)	274 (5)
Birth setting‡	162 (54)	NA	NA	NA	NA
Severity					
Hospitalization§	253 (84)	36 (97)	1,064 (95)	3,535 (95)	4,635 (92)
Death or fetal loss¶	58 (19)	0# (0)	113# (10)	545# (14)	658# (13)

*NA, not applicable †When *Listeria monocytogenes* is isolated from multiple anatomic sites, only a single site is reported (priority order: cerebral spinal fluid, blood, other sterile site, and birth setting). ‡Either from a newborn, fetus, stillborn or from maternal tissue (placental tissue, uterus, cervix). §Hospitalizations among singleton neonates for 224 pregnancy-associated cases. ¶26 neonatal deaths, 32 fetal losses. Among all pregnancy-associated cases 161 premature births were recorded. #Information available for 4,989/5,064 (99%) of notified cases.

## Conclusions

The aging of the population of Germany as a result of demographic shifts that will continue in the coming years may partially explain the increase in listeriosis cases and the median age of patients. In addition, factors related to the foodborne nature of the disease and an increase in exposure to *Listeria* must be presumed; it is possible that people eat more RTE food or that RTE food is more likely to become contaminated, although only single-case findings of *L. monocytogenes* >100 CFU/g have been detected in RTE foods in recent years (*8*). 

The additional case numbers in some quarters of the year (Figure 1) were all associated with large-scale outbreaks (*3*,*6*). Successfully identifying and controlling large outbreaks, especially after whole-genome sequencing–based surveillance was introduced, possibly explains why the trend in increases ended after 2017 (*9*). Overall listeriosis incidence in Germany is higher than in all neighboring countries except Denmark (*10*). In Europe, incidence is generally higher in countries in Scandinavia and the Baltic region and lower in the United Kingdom and Ireland (*10*).

As is the case for other pathogens, listeriosis surveillance results in underascertainment, although it is difficult to quantify by how much. *Listeria* sepsis cannot be clinically distinguished from other bacterial sepsis, and isolating *Listeria* or detecting DNA from blood samples is often impossible because bacteremia is absent or intermittent. In addition, laboratory diagnostic testing is often not performed after abortions or stillbirths or for persons who are found dead. 

Listeriosis has one of the highest case-fatality rates among notifiable infectious diseases. The case-fatality rate for Germany in this study is surprisingly lower than that for Europe overall, 15.6% (*10*), and for the United States, 21% (*11*). A cohort study in France reported a 3-month death rate of 45% for bacteremia from *Listeria* infection and 30% for neurolisteriosis cases (*12*). Lower rates may be partially explained by well-equipped intensive care units, but it is more likely that many deaths occurring long after original disease notifications were not reported to public health departments. 

Of interest, surveillance data from the United States indicate more listeriosis among women and higher proportions of pregnancy-associated cases (*11*,*13*) than in our study. One explanation might be that, in Germany, meat products, more often eaten by men, constitute prominent outbreak vehicles (*3*,*4*,*6*,*7*), whereas in the United States several outbreaks were caused by nonanimal products or cheese (*11*).

Systematic whole-genome sequence typing of *Listeria* isolates from patients would aid in detecting and investigating outbreaks. These molecular data should be integrated into surveillance data from cases notifications and isolates found in food. Combining data from molecular surveillance with epidemiologic investigations would help systematically identify and eliminate contaminated sources, which might have the greatest effect on reducing the overall burden of listeriosis and thus flattening its high incidence curve. Two factors interact to have the greatest influence on personal risk profiles. Listeriosis is highly associated with age, which is affirmed in our study, and strongly associated with documented immunosuppressive conditions (*2*). Persons with these risk profiles should be targeted in information campaigns about how to safely consume RTE foods and avoid certain types of cheeses, meat products, and smoked or graved (cured) fish products. All food producers, and especially those providing food for immunocompromised patients in healthcare facilities, should take steps to minimize *L. monocytogenes* hazards when producing, selecting, and preparing food. 

Appendix Additional information about listeriosis incidence and case-fatality rates in Germany, 2010–2019
