# Supplementary material for: Ongoing High Incidence and Case-Fatality Rates for Invasive Listeriosis, Germany, 2010–2019
Source: Emerg Infect Dis. 2021 Sep;27(9):2485–8. doi: 10.3201/eid2709.210068 (PMC8386774; doi:10.3201/eid2709.210068)
Supplement: Appendix — Additional information about listeriosis incidence and case-fatality rates in Germany, 2010–2019 [file 21-0068-Techapp-s1.pdf]

Article DOI: https://doi.org/10.3201/eid2709.210068

Ongoing High Incidence and Case-Fatality Rates for Invasive Listeriosis, Germany, 2010–2019

Appendix

Description of listeriosis surveillance in humans in Germany

According to the German Protection against Infection Act, laboratory confirmation of *Listeria monocytogenes* isolation or detection of nucleic acids from blood, cerebrospinal fluid (CSF), or other usually sterile sites is notifiable to local health authorities. These agencies complete and verify clinical information by adding data derived from interviewing patients or their physicians. In neonates, the isolation or detection of nucleic acids of *L. monocytogenes* is notifiable and fulfills the listeriosis case definition independent of clinical signs and symptoms of the newborn and mother. Data on patients fulfilling criteria for the reference definition are electronically transmitted to the federal state health authorities and from there to the Robert Koch Institute (RKI) (1). The reference definition for listeriosis (2) includes clinical criteria (fever, influenza-like symptoms, sepsis, meningitis, meningoencephalitis, or local infections) and laboratory criteria (isolation of *L. monocytogenes* or detection of nucleic acid from *L. monocytogenes* in blood, CSF, or another normally sterile site). Before the third quarter of 2015, two groups of patients were not included in the reference definition: those with unknown or unfulfilled clinical criteria and those with nucleic acid detection only. Data from these groups are displayed separately in Figure 1 in the main article (dark blue part of bars) to make changes in trends over time more apparent. The German surveillance system counts mother and child as 2 separate cases on the basis of time and place of infection. In contrast, for the analyses of clinical characteristics and isolate sources mother-child pairs are counted as 1 pregnancy-associated case. This led to discrepancies in total numbers between the figures and tables.

When patients in notified cases die, local health authorities specify the circumstances of death, including whether the patient died directly or indirectly from listeriosis. The information on the death certificate could then be used to assess the circumstances of death.

Unless listeriosis was mentioned as part of the causal chain leading to death, “other cause” would be selected as cause of death.

The consultant laboratory for *Listeria* at RKI collects and performs whole genome sequencing (WGS) on clinical *L. monocytogenes* isolates from human infections in Germany. Unlike in the reportable disease notification system, submission of clinical *L. monocytogenes* isolates from primary laboratories to the RKI consultant laboratory is voluntary but is encouraged by public health authorities. Genome sequence information from isolates in individual cases in *L. monocytogenes* clusters or outbreaks can thus be linked with notified listeriosis cases throughout Germany. In case of outbreak detection by WGS, mandatory notification data from listeriosis cases and typing data for the *L. monocytogenes* isolates that were sent to the RKI can be merged for investigation.

Subtyping of *L. monocytogenes* isolates from food samples was performed at the National Reference Laboratory for *Listeria monocytogenes* at the German Federal Institute for Risk Assessment (BfR) in Berlin. The consultant laboratory and the BfR regularly compare *L. monocytogenes* strain identities from clinical and food isolates.

The information on outbreaks is submitted to the federal state and local public health departments. After detection, cross-regional listeriosis outbreaks are jointly investigated by the public health services as well as food safety agencies of the districts, cities, federal states, and at the national level. Listeriosis surveillance in Germany is limited in the sense that food consumption histories are only collected after detection of outbreaks. Patients or their relatives who consent are interviewed using a standard comprehensive exploratory questionnaire. If a certain food is already indicated as the probable vehicle, a shorter, more focused questionnaire is used for the interview. Furthermore, several listeriosis outbreak investigations require international collaboration, including with the European Center for Disease Prevention and Control (ECDC) and its Epidemic Intelligence Systems. In parallel, food safety agencies (e.g., European Food Safety Authority) play important roles on all levels of the federal system to safeguard necessary investigations and interventions. The OneHealth approach has been incorporated by stakeholders for foodborne disease surveillance and control in Germany. Data on the occurrence of zoonotic pathogens and related antimicrobial resistance in food has been collected by food safety authorities of the federal states since 2009 as part of official food and veterinary surveillance guidelines. Information is evaluated and published annually (3).

Description of listeriosis outbreaks relevant for Germany

In recent years, several protracted and geographically widespread listeriosis outbreaks have been identified in Germany through the systematic application of bacterial typing identified by WGS in the context of disease surveillance. An outbreak with 79 cases occurring during 2012–2016 (4), predominantly in south Germany, could be successfully traced back to meat products from a specific company by systematic typing of *L. monocytogenes* isolates (5). Another listeriosis outbreak with 83 cases during 2013–2017 was associated with meatballs (6), a source pinpointed by patient interviews and confirmed by WGS. In the course of this outbreak investigation, 2 distinct genotypes turned out to be involved. One of the largest European outbreaks included 112 notified listeriosis cases throughout Germany and happened during 2018–2019. A case-control study followed by targeted household food sampling of listeriosis patients identified blood sausage from 1 supermarket chain as a possible vehicle. The association between the outbreak cases and the blood sausage was confirmed by WGS (7). In 2019, there was a nationwide outbreak with 39 cases primarily in healthcare facilities. Epidemiologic and food tracing investigations in healthcare facilities enabled the detection of contaminated meat products from a manufacturer that provided catering for hospitals (8). The detection of this company as the source led to a large-scale product recall and was accompanied by extremely broad media coverage.

In 2021, BfR and RKI published a report on 22 listeriosis outbreaks in Germany in recent years that focused on the role of salmon or salmon products (9). There were a total of 218 cases reported. Forty-four case-patients died, 17 from listeriosis; 4 cases were pregnancy-associated. The clinical isolates from the 22 outbreaks identified by WGS are not genetically related to each other but each outbreak closely related to isolates from different salmon products (9).

Federal states in eastern Germany show higher incidence compared with those in western Germany (Appendix Figure). The lowest average annual incidence was seen in Saarland (0.48/100,000) and the highest in Saxony (1.28/100,000). The geographical pattern of listeriosis distribution in Germany remains largely unexplained. Recently identified listeriosis outbreaks had regional patterns most likely determined by retail supply organization but outbreak areas were not concordant with high-incidence areas.

Even though these prominent outbreaks highlight meat and salmon products as vehicles for listeriosis outbreaks, it should be kept in mind that other food items, particularly cheeses, sliced fruits, and prepared salads, might cause outbreaks or sporadic cases.

International outbreak investigations provide evidence for fruits (10,11), sprouts (12), ice cream (13), and pasteurized dairy products (14,15), but also raw dairy products (16,17), fish (18–20) and frozen products (21) as sources, showing the wide range of possible listeriosis outbreak vehicles. The largest known listeriosis outbreak in world history took place in South Africa in 2017–2018 and was caused by the processed RTE meat product polony, a smoked sausage typically made of pork and beef (22). Of the 937 laboratory-confirmed cases, 465 (50%) were pregnancy-associated (22).

Appendix References

1. Federal Ministry of Justice and Consumer Protection. Law for the prevention and control of infectious diseases in humans [German]. [cited 2021 June 08] https://www.gesetze-im-internet.de/ifsg/
2. Robert Koch-Institut. Case definitions of the Robert Koch Institute for the transmission of illnesses or deaths and evidence of pathogens [German]. [cited 2021 June 08] www.rki.de/falldefinitionen
3. Bundesamt für Verbraucherschutz und Lebensmittelsicherheit (BVL). Zoonoses monitoring [German]. [cited 2021 June 08] https://www.bvl.bund.de/DE/Arbeitsbereiche/01_Lebensmittel/01_Aufgaben/02_AmtlicheLebensmittelueberwachung/06_ZoonosenMonitoring/lm_zoonosen_monitoring_node.html
4. Ruppitsch W, Prager R, Halbedel S, Hyden P, Pietzka A, Huhulescu S, et al. Ongoing outbreak of invasive listeriosis, Germany, 2012 to 2015. Euro Surveill. 2015;20.
5. Kleta S, Hammerl JA, Dieckmann R, Malorny B, Borowiak M, Halbedel S, et al. Molecular tracing to find source of protracted invasive listeriosis outbreak, southern Germany, 2012–2016. Emerg Infect Dis. 2017;23:1680–3.
6. Lüth S, Halbedel S, Rosner B, Wilking H, Holzer A, Roedel A, et al. Backtracking and forward checking of human listeriosis clusters identified a multiclonal outbreak linked to *Listeria monocytogenes* in meat products of a single producer. Emerg Microbes Infect. 2020;9:1600–8.
7. Halbedel S, Wilking H, Holzer A, Kleta S, Fischer MA, Luth S, et al. Large nationwide outbreak of invasive listeriosis associated with blood sausage, Germany, 2018–2019. Emerg Infect Dis. 2020;26:1456–64.
8. Lachmann R, Halbedel S, Adler M, Becker N, Allerberger F, Holzer A, et al. Nationwide outbreak of invasive listeriosis associated with consumption of meat products in health care facilities, Germany, 2014–2019. Clin Microbiol Infect. 2020;27:P1035.E1–5.

9. Bundesinstitut für Risikobewertung, Robert Koch-Institut. Several outbreaks of listeriosis in Germany with evidence of smoked or pickled salmon products as the cause of infections [German]. Epid Bull. 2021;3:3–9.

10. Jackson BR, Salter M, Tarr C, Conrad A, Harvey E, Steinbock L, et al. Notes from the field: listeriosis associated with stone fruit—United States, 2014. MMWR Morb Mortal Wkly Rep. 2015;64:282–3.

11. McCollum JT, Cronquist AB, Silk BJ, Jackson KA, O'Connor KA, Cosgrove S, et al. Multistate outbreak of listeriosis associated with cantaloupe. N Engl J Med. 2013;369:944–53.

12. Farber JM, Carter AO, Varughese PV, Ashton FE, Ewan EP. Listeriosis traced to the consumption of alfalfa tablets and soft cheese. N Engl J Med. 1990;322:338.

13. Centers for Disease Control and Prevention. Multistate outbreak of listeriosis linked to Blue Bell creameries products (final update). 2015. [cited 2021 June 08] http://www.cdc.gov/Listeria/outbreaks/ice-cream-03-15

14. Fretz R, Sagel U, Ruppitsch W, Pietzka A, Stoger A, Huhulescu S, et al. Listeriosis outbreak caused by acid curd cheese Quargel, Austria and Germany 2009. Euro Surveill. 2010;15.

15. Koch J, Dworak R, Prager R, Becker B, Brockmann S, Wicke A, et al. Large listeriosis outbreak linked to cheese made from pasteurized milk, Germany, 2006–2007. Foodborne Pathog Dis. 2010;7:1581–4.

16. Linnan MJ, Mascola L, Lou XD, Goulet V, May S, Salminen C, et al. Epidemic listeriosis associated with Mexican-style cheese. N Engl J Med. 1988;319:823–8.

17. MacDonald PDM, Whitwam RE, Boggs JD, MacCormack JN, Anderson KL, Reardon JW, et al. Outbreak of listeriosis among Mexican immigrants as a result of consumption of illicitly produced Mexican-style cheese. Clin Infect Dis. 2005;40:677–82.

18. European Centre for Disease Prevention and Control. Multi-country outbreak of *Listeria monocytogenes* clonal complex 8 infections linked to consumption of cold-smoked fish products. 2019 [cited 2021 June 08]. https://www.ecdc.europa.eu/en/publications-data/multi-country-outbreak-listeria-monocytogenes-fish-products

19. Gillesberg Lassen S, Ethelberg S, Bjorkman JT, Jensen T, Sorensen G, Kvistholm Jensen A, et al. Two *Listeria* outbreaks caused by smoked fish consumption using whole-genome sequencing for outbreak investigations. Clin Microbiol Infect. 2016;22:620–4.

20. Schjorring S, Gillesberg Lassen S, Jensen T, Moura A, Kjeldgaard JS, Muller L, et al. Cross-border outbreak of listeriosis caused by cold-smoked salmon, revealed by integrated surveillance and whole genome sequencing (WGS), Denmark and France, 2015 to 2017. Euro Surveill. 2017;22.

21. European Centre for Disease Prevention and Control. Rapid risk assessment: multi-country outbreak of *Listeria monocytogenes* serogroup IVb, multi-locus sequence type 6, infections linked to frozen corn and possibly to other frozen vegetables—first update. 2018. [cited 2021 June 08] https://www.ecdc.europa.eu/en/publications-data/rapid-risk-assessment-multi-country-outbreak-listeria-monocytogenes-serogroup-ivb

22. Thomas J, Govender N, McCarthy KM, Erasmus LK, Doyle TJ, Allam M, et al. Outbreak of listeriosis in South Africa associated with processed meat. N Engl J Med. 2020;382:632–43.

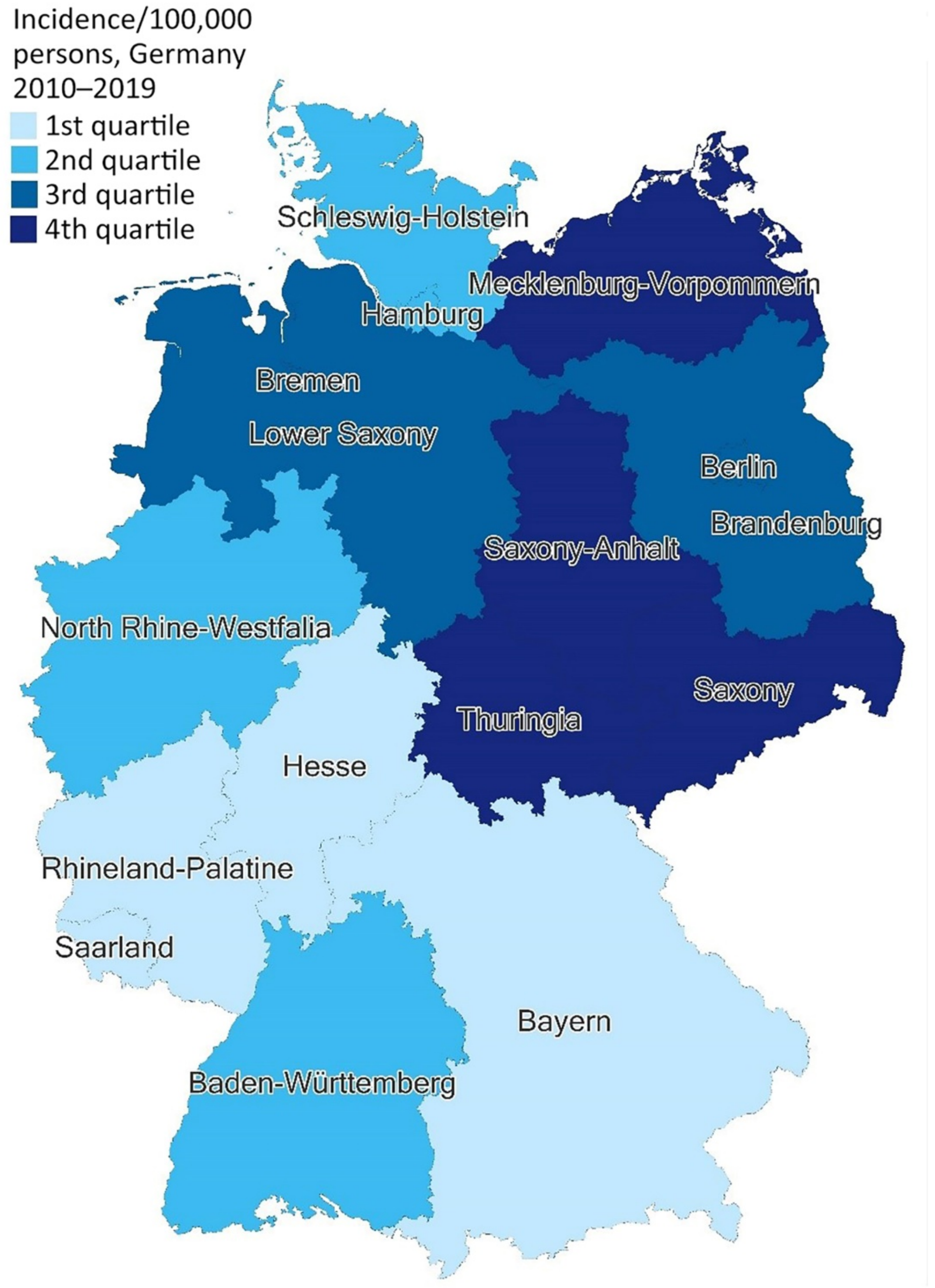

Appendix Figure. Spatial distribution of average annual incidence of pregnancy-associated and non–pregnancy associated listeriosis in federal states, Germany, 2010–2019 (n = 5,575)
